# Participants’ experiences of the benefits, barriers and facilitators of attending a community‐based exercise programme for people with chronic obstructive pulmonary disease

**DOI:** 10.1111/hsc.12929

**Published:** 2019-12-13

**Authors:** Oluwasomi F. Meshe, Hilary Bungay, Leica S. Claydon

**Affiliations:** ^1^ Faculty of Health, Education, Medicine and Social Care Anglia Ruskin University Chelmsford Essex UK; ^2^ Faculty of Health, Education, Medicine and Social Care Anglia Ruskin University Cambridge Essex UK

**Keywords:** community‐based exercise programme, COPD, rehabilitation

## Abstract

Community‐based exercise programmes (CEPs) are aimed at sustaining benefits of pulmonary rehabilitation (PR) in people with chronic obstructive pulmonary disease (COPD). The aim of this study was to understand participants’ experiences of the benefits, barriers and facilitators of adherence to a CEP. A descriptive qualitative design was applied, employing in‐depth semi‐structured interviews with a convenience sample of 12 participants with COPD attending a CEP in the East of England. Interviews were audio‐recorded, transcribed and analysed using thematic analysis. Four main themes were identified: perceived benefits, enablers and barriers, perception of safety and recommendations for programme improvement. Participants experienced physical, social and psychological benefits. Regular attendance is important to avoid deterioration in perceived benefits. CEPs may therefore provide a cost‐effective approach to improving and sustaining initial benefits of PR. Enablers included ease of access, perceived benefits and convenient programme components, being a retiree, social support and seasons. Identified barriers to attendance were poor physical health, family commitments and transport difficulties. The findings clearly suggest that a CEP supervised by an exercise instructor motivates participants to attend and exercise regularly. Increasing adherence to an exercise programme will prevent deterioration in perceived health, in addition to the physical, psychological and social benefits to the individual.

**What is known about this topic**

- The robust benefits of pulmonary rehabilitation programmes in people with chronic obstructive pulmonary disease (COPD) are short‐lived.
- Sustaining benefits of PR is a global priority.
- Community‐based exercise programme (CEPs) improve quantitative measures of exercise capacity and health‐related quality of life in people with COPD.

**What this paper adds**

- Participants perceive regular attendance at CEPs helps them avoid deterioration in physical ability and COPD symptoms as well as providing social and psychological benefits.
- Exercising in a group enabled participants to adhere to exercise.
- CEPs should consider barriers to participation such as physical health issues, transport and timing. Exercise instructors can address safety concerns and support in a CEP.

## 1 INTRODUCTION

Pulmonary rehabilitation (PR) remains the main non‐pharmacological strategy for managing people with chronic obstructive pulmonary disease (COPD) (Vestbo et al., 2013). It combines exercise training, education, psychological counselling and social support to improve outcomes such as levels of daily physical activity, exercise capacity, health‐related quality of life (HRQoL), dyspnoea and duration of hospital admission (Egan et al., 2012; Ries et al., 2007; Seymour et al., 2010). However, most PR programmes (PRPs) last 4–12 weeks and research evidence suggest that benefits start to diminish from 4 weeks after programme completion (Karapolat et al., 2007), with progressive decline and return to baseline values after 12 months (Egan et al., 2012; Holland et al., 2017). Consequently, the clinical guidelines of the Global Initiative for Chronic Obstructive Lung Disease, GOLD (Vestbo et al., 2013) and the most recent official statement of European Respiratory Society (ERS) (Watz et al., 2014) advised rehabilitation professionals to refer graduates of PRPs to home and/or community‐based exercise programmes (CEPs) to further improve and/or sustain benefits from initial PR.

Non‐adherence to advice from professionals to continue exercising at home following PR was identified as the major reason for decline in benefits (Beauchamp, Evans, Janaudis‐Ferreira, Goldstein, & Brooks, 2013). This has stimulated interests in CEPs. A meta‐analysis concluded that, compared to usual care, supervised CEPs maintained participants’ exercise capacity for 6 months (Beauchamp et al., 2013). However, this benefit was not maintained after 12 months and there was no significant improvement in HRQoL. Consequently, the authors highlighted the need for further studies to explore facilitators and barriers in order to optimise adherence to CEPs. This is consistent with the recommendations by a multidisciplinary Task Force of experts representing the ERS (Watz et al., 2014).

Reduced levels of PA were found to be the strongest predictors of all‐cause mortality in people with COPD (Waschki et al., 2011). Increasing levels of PA is one of the goals of COPD management (Vestbo et al., 2013; Watz et al., 2014). However, maintaining consistent levels of daily PA following PR is a challenge for people with COPD due to on‐going perception of breathlessness, which can potentially lead to reversion to a vicious cycle of inactivity (Troosters et al., 2013). Graduates of PRPs expressed desire to remain connected to their peers, health professionals and be facilitated into a structured and supervised CEP in order to be more physically active in daily life (Hogg, Grant, Garrod, & Fiddler, 2012). A recent mixed‐methods systematic review highlighted some factors that hindered and enabled individuals to attend CEPs (Meshe, Claydon, Bungay, & Andrew, 2017). Healthcare professionals, social support, goal setting, reduced fear, seeing benefits, availability of different exercise modalities facilitated attendance and increase in levels of PA. Factors such as lack of social support, fear, changing physical health (co‐morbidities and exacerbations) and weather hindered participation.

Participants’ views of the benefits, barriers and enablers of participation in a long‐term exercise programme have been recognised as important aspects of COPD management (Vestbo et al., 2013). These provide useful information when considering how to design novel interventions for improving and maintaining benefits of PRPs and for understanding how to make them more successful and sustainable. They also provide insight into how rehabilitation specialists can help people with COPD translate benefits of exercise intervention into being more physically active in daily life and optimising their quality of life (Sabit et al., 2008; Watz et al., 2014). The majority of previous studies have focused on investigating the effectiveness of post‐rehabilitation CEPs using quantitative study designs. The outcomes of interest were levels of daily activities, exercise capacity, HRQoL, dyspnoea and number of hospital admission (Beauchamp et al., 2013; Egan et al., 2012; Seymour et al., 2010).

The study reported here is part of a sequential mixed methods study with a quantitative and qualitative phase. In the quantitative phase daily physical activity, health status, exercise capacity, pulmonary function and number of hospital admissions were recorded at two time points (start of the study and three months later) in 30 participants completing a CEP. The relationship between daily physical activity and these clinical outcomes was investigated (Meshe, 2018). This paper relates to the qualitative phase of the study. To obtain an understanding of participants’ views of CEP a nested qualitative study was conducted (Creswell & Clark 2011). Semi‐structured interviews were undertaken with a sub‐sample of 12 participants. The interviews were guided by two research questions: (a) what are participants’ views of the benefits of a CEP? (b) What helps participants to be able to attend the weekly exercise classes and what makes it more difficult for them to attend? The findings have implications for clinical practice in understanding how to improve quality of programmes and thus promote long‐term adherence.

## 2 METHODS

### 2.1 The community‐based exercise programme

A local district council developed the CEP in partnership with a lifestyle and facilities management company. The programme was designed to improve the general well‐being of people aged (≥55 years) with long‐term health conditions. It was offered at two community recreation centres in the East of England. A team of physiotherapists and respiratory nurses from a local hospital referred people with COPD to the CEP after completing an 8‐week hospital‐based PR.

The exercise programme was supervised by an exercise instructor, registered with the regulatory body the ‘Register of Exercise Professionals’ (://www.exerciseregister.org). To our knowledge, this is quite novel. Most if not all PR and CEPs are supervised by healthcare professionals, registered physiotherapists and respiratory nurses (Beauchamp et al., 2013; Desveaux, Janaudis‐Ferreira, Goldstein, & Brooks, 2015). Exercise training includes breathing exercises, 24‐seated warm‐up and cool‐down exercises, 5 cardiovascular and 12 resistance‐training exercises. These are similar to those reported in most PRPs (Desveaux et al., 2015; Watz et al., 2014), which have robust benefits for people with COPD (Ries et al., 2007). Each session lasted approximately 90 min and participants attend twice per week. The specific exercise modalities, intensity, frequency and duration are matched to an individual's capacity and specific needs. Twenty minutes of social time is embedded in the session to enable participants to interact, and share snacks and soft drinks. The CEP did not include any education or advice on activities outside the group. However, participants interacted with one another and discussed different issues.

### 2.2 Design

A descriptive qualitative design was used and semi‐structured interviews with a convenience sample of 12 participants were conducted. All interviews were audio‐recorded, transcribed and thematically analysed. The University's ethics panel and The Social Care Research Ethics Committee (SCREC) approved the protocol (reference number 15/IEC08/0034), and the local council approved the study following review of all the SCREC and University approval documentation.

Key ethical concerns were managed appropriately. Risks of breathlessness, fatigue, discomfort and falls associated with exercising were managed by an instructor, who designed the moderate‐intensity exercises, supervised all sessions and ensured they were performed intermittently (3 min of exercise interspersed with 3 min of rest) as suggested by Sabapathy, Kingsley, Schneider, Adams, and Morris (2004). He also supported participants with using exercise equipment and ensured they wore appropriate attire. Psychological discomfort, which is common in qualitative research (Sanjari, Bahramnezhad, Fomani, Shoghi, & Cheraghi, 2014) was minimised by ensuring that the interview questions did not relate to any sensitive personal issues. The Interviews were conducted in a safe, conducive and private room in a leisure centre. Participants’ confidentiality was protected by physically moving audio recorders directly from interview room to the first author's office. The recorders were stored in a filing cabinet. We anonymised and stored transcripts of interviews in a password‐protected laptop.

### 2.3 Participants and recruitment

Participants for the overall study were recruited from a CEP. The first author attended two exercise training sessions to introduce the study, put up research flyers and distribute information leaflets. Prospective participants were given two weeks between providing information and contacting the first author so they could consider fully whether they wanted to participate in the research. Those who responded by contacting the first author were scrutinised for eligibility: (a) clinically diagnosed with COPD based on GOLD criteria (Vestbo et al., 2013), FEV1/FVC <0.7 and FEV_1_ ≥80%, (b) previously completed primary PR (c) willing and able to provide consent (d) clinically stable and able to communicate effectively in English language (e) attended the programme for at least 2 months. Exclusion criteria were any known hearing or other physical problems that could prevent effective collaboration. Eligible participants were asked for their verbal and written consent. In total, 30 people consented to take part in the study and following the quantitative phase of the study (Meshe, 2018), participants were invited to be interviewed. Twelve participants consented to this second phase of the research.

### 2.4 Data collection

The interviews were conducted by the first author between April–May 2016, using an interview guide, which was adapted from Desveaux, Beauchamp, Rolfe, Goldstein, and Brooks (2014) (see Table 1). Additional questions and probes were used to clarify issues that emerged during the interviews. Each interview was audio‐recorded and lasted for about 40 min. A test interview was conducted to get a general feel for how the interviews would go. This did not alter any part of the interview schedule. It enabled the interviewer to estimate the duration of each interview and troubleshoot unforeseen issues relating to the digital audio‐recording device. The first author knew these questions were fit for purpose as participants demonstrated full understanding and responded appropriately to the questions without deviating from the main issues under discussion. The interviewer was a doctoral student. Although, new to qualitative interviewing he was trained to undertake this role and was supervised by the second author, an experienced qualitative researcher.

**Table 1:** The interview guide

Topic	Questions (for participants)
Benefits	Do you think this follow up class would have any health benefits for you? Do you think the program is beneficial? Why or why not? What do you like best about the programme? (d) What do you like least about the program?
Safety	Do you feel safe while exercising in the community centre? What made you feel safe while exercising in the community centre? Did anything make you feel unsafe? (Probe: monitoring equipment, connection with rehab centre)
Barriers and Facilitators	What helped you to be able to attend the community programme? What made it more difficult for you to attend? (Probe: programme elements such as time of day, duration, proximity, personal factors such as health, supportive relationships, etc.)
Recommendations for improvement	What would you recommend we keep the same and what would you recommend we change? (Probes: frequency, length, time of day of the programme)

Note

Adapted with permission from: Desveaux et al. (2014).

### 2.5 Data analysis

The interview transcripts were thematically analysed using the six‐phase approach advanced by Braun and Clarke (2006). The interview guide was designed to answer specific research questions regarding the perceived benefits and facilitators and barriers to the programme, and therefore a deductive approach to analysis was adopted. However analysis was also inductive because it aimed to respond to new themes that were identified from the data. The primary aim was to identify themes, which were the repeating ideas in the texts (Clarke & Braun, 2013). To ensure credibility and rigour, the first and second authors independently coded the transcripts and identified themes. The recurring themes were identified based on their frequency in the transcripts. The research questions of the study were used to guide this process and therefore benefits, facilitators and barriers to participation were captured.

**Figure 1:**
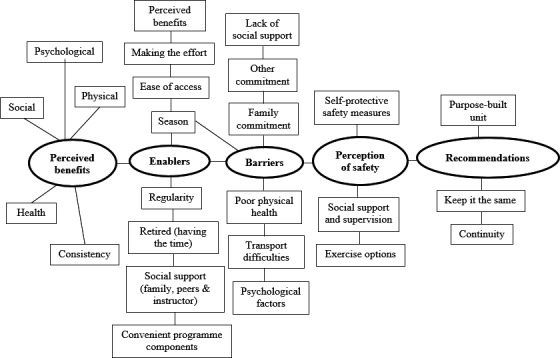
The themes and subthemes from interview data

Following initial coding, the coded structures and themes that were independently generated were compared, discussed and refined until they reached consensus. The third author was also consulted during data interpretation to resolve any discrepancies. This peer review approach was employed to minimise bias and enhance trustworthiness of findings (Krefting, 1991). A thematic map (Figure 1) was generated to illustrate the overall relationship between themes. Direct quotations were used to represent categories and enable participants’ voices to come through (Creswell, 2013). Pseudonyms were used throughout to maintain confidentiality of participants.

## 3 FINDINGS

### 3.1 Participants

The demographic characteristics (including education, employment, years enrolled in the CEP and treatment) of the 12 participants interviewed are summarised in Table 2 and to some extent, correspond to most features of people with COPD (e.g. age range and smoking status) (Meyer, Mannino, Redd, & Olson, 2002; Jones et al., 2014). The sample consisted of seven women and five men. All were White British and retired. On average, participants attended 83% of all 24 scheduled exercise sessions.

**Table 2:** Characteristics of the participants

ID	Sex	Age	Marital status	BMI	Ethnic group	Education status	Employment status	Smoking status	FEV1 (% predicted)	Years in CEP	Proximity to CEP (miles)	Attendance (total = 24 sessions)
P1	F	89	Single	18.2	White	Prof qual.	Retired	FS	75	<1	7	15
P5	F	78	Married	28.44	White	Prof qual.	Retired	OS	78	1‐3	10	24
P7	F	77	Married	34.24	White	GCSE/O'Level	Retired	FS	57	<1	1	24
P10	F	73	Widowed	27.74	White	No qual.	Retired	OS	54	<1	4	22
P11	F	72	Widowed	30.74	White	Other qual.	Retired	FS	83	>3	1	18
P12	F	72	Married	38.82	White	Other qual.	Retired	FS	78	1‐3	5	23
P15	F	69	Married	28.25	White	Other qual.	Retired	FS	51	1‐3	1	23
P17	M	79	Married	34.8	White	Prof qual.	Retired	FS	78	1‐3	8	18
P19	M	73	Single	39.52	White	Other qual.	Retired	FS	66	>3	12	19
P20	M	72	Married	23.25	White	Degree+	Part‐time	Never	34	1‐3	11	19
P22	M	70	Married	32.32	White	O & A Levels	Retired	FS	55	1‐3	4	18
P23	M	68	Married	26.94	White	GCSE/O'Level	Retired	FS	80	<1	9	22
Mean		74.33		30.27					66		6	20 (83%)

Abbreviations: BMI, body mass index; CEP, community‐based exercise programme; F, female; FEV1, forced expiratory volume in 1 s; FS, former smoker; GCSE, general certificate of secondary education; M, male; No qual., no qualification; OS, occasional smoker; O & A, ordinary and advance level, O Level, ordinary level; Prof qual., professional qualification.

In the following section, the main themes shown in Figure 1 perceived benefits, enablers and barriers, perception of safety; and recommendations for improvement are presented. Here benefits are improvements participants associated with attending the programme. Enablers are factors that facilitated or helped participants to attend the programme and barriers are factors that hindered or did not help programme attendance.

## 4 PERCEIVED BENEFITS

The participants valued the CEP and emphasised its benefits, describing these under five sub‐themes: health, physical, psychological, social benefits and consistency.

### 4.1 Health benefits

Participants overwhelmingly described their experiences of health benefits in terms of the perceived improvement in general well‐being, and more specifically absence of chest infections, coping with breathlessness, getting off antibiotics, improved breathing and the feeling that COPD had not progressed or got any worse:

I think I was diagnosed with COPD in about 2013 or something. I just don't feel that it's gone worse. I mean I feel it hasn't progressed or get any worse at all, and if anything, I feel more comfortable now. In fact, I had a spirometer test last week, and it was as good as it was three years ago, and in some respect even slightly better. So I’m sure the exercise programme has helped in that respect (Bill, P20).

The programme was seen to have a positive impact on health by improving overall well‐being and improving breathing, as well as ameliorating the negative effects of COPD such as risks of chest infections and feelings of breathlessness.

### 4.2 Physical benefits

At a functional level, participants described their experience of strengthened skeletal muscles and this had the added benefits of increased physical fitness and functioning. For most participants, the programme was their first experience of exercising in a gym. They related feeling stronger, fitter and better compared to when they were not going to the gym:

I mean I never did [exercises]── you know, just the fact of coming to an exercise class── to the gym twice a week is better than it was. I didn't use to do that. And── and I feel physically stronger and better (Lucy, P11).

Participants also described improvement in overall physical functioning in terms of improvement or maintenance of their activities of daily living, positive feedback from friends and family members regarding their physical condition, and personal satisfaction with their abilities to use gym equipment, which they had previously found difficult to use. One participant explains:

I’ve been coming here for── just under 2 years and I know I’ve improved in the gym. I mean, when I first came here there is no way I could have got on that Cross Trainers, no way. So I know I have improved and I go walking with a friend and some days── if the weather is good, she will say to me, “You are walking quicker”. So── you know, maybe I haven't noticed it but other people have noticed it (Zoe, P15).

Beside the several references to performance and maintenance of levels of different physical activities, participants also appreciated the restorative nature of the programme because they had been able to return to hobbies and activities they used to engage in before they were restricted by COPD.

### 4.3 Social benefits

The group nature of the exercise training sessions provided positive social benefits, giving participants the opportunity to meet people with similar conditions. The camaraderie engendered in the group was well regarded and it was felt to have been of immense benefit to them. It was recognised that being part of a group made them realise that they are not alone and other people experience similar problems, this positively influenced mutual care and support.

It's very good, being a group thing and meeting people who are similar to yourself. So you don't feel embarrassed or anything like that. You feel on a par. You're not thinking, “Oh, I can't do this” because everybody else is in the same boat as you (Monica, P10).

### 4.4 Psychological benefits

A sense of improved psychosocial well‐being was an important benefit of the programme. This was described in terms of reduced depression, a reduction in fear/anxiety associated with being breathless when performing exertional activities and a positive impact of improved motivation and self‐confidence. Participants linked these benefits to the social aspect of the programme, which in addition to the camaraderie of the group provided the opportunity to get out of their houses, meet and talk to people as well as participate in activities.

I do suffer with depression. I am on medication for depression. But you see, it's important to come here really because that does help the depression. Coming here definitely helps me overcome depression. Because── I think talking to people, getting different people's points of view, I think it── it definitely helps, yeah (Merlin, P19).

Over half of the participants, reported having some exercise equipment at home but did not use them because it was difficult to be motivated at home. They reported though that being with the group provided intrinsic motivation and confidence to do more exercises than they would if they were left on their own.

I think if I was left on my own devices I just wouldn't do it. If I didn't have to come here, I don't think I would be doing the exercises at home. No way! (Zoe, P15).

### 4.5 Consistency

Participants acknowledged the need for attendance to prevent deterioration in physical ability and an increase in COPD symptoms. Those whom had stopped attending the exercise classes for any reason, experienced deterioration and recognised the need for regular attendance in order to avoid future setbacks:

One thing I do notice with this── I was recently [sic] had two weeks, no three to four weeks not coming and for whatever reason. I noticed it when I came back that I can't do what I was doing before. So it's very important that you have a consistency. As soon as you don't go in once── or if you leave it for a week or two, the body falls back. You mustn't take the time off that's what I noticed. It's not good, too much time off, you go backward (Brendan, P23).

## 5 ENABLERS AND BARRIERS TO ATTENDANCE

Attending sessions could sometimes present challenges for participants but there were also factors that enabled attendance.

### 5.1 Enablers

To attend regularly participants described six enablers of attendance, including ease of access, perceiving benefits to attending, structured and convenient programme components, being retired, social support and the summer season. It was important that the programme was convenient with regards to time of day, day of week, duration of exercise training and in a venue that is close to where they live and/or accessible by car. Being retired allowed participants to have time to attend, and this may reflect the fact that all but one of the participants described themselves as retired. People were encouraged to attend because they experienced the benefits of the programme. Support from family members, peers and the exercise instructor were also considered important. Participants expressed how at times weather influenced their well‐being and attendance of exercise classes, winter was associated with worse health, whereas summer it was easier to attend as their health was generally better and they felt more motivated:

I did come for a couple of times── twice in the week in the summer. Everything is harder in the winter for me. Arthritis is worse in the winter. Hopefully, as the weather gets better now I want to try and do two days a week again because I’m better in the summer (Monica, P10).

### 5.2 Barriers

The barriers to attendance included their own poor physical health, family commitments and transport difficulties. Poor physical health was the most frequently cited factor that affected attendance and was related to experiences of respiratory and urinary infections and arthritis.

[Pause]── well I think── I mean the only reason I guess, I didn't come for a couple of weeks, because── because I wasn't too well. If I had you know,── sometimes if I get like── chest infection, something like that, then I won't come. I mean nothing in particular will stop me coming, except if I was not well (Bill, P20).

Participants often prioritised family commitments over attending exercise classes, such as caring for physically unwell partners and attending to grandchildren. The distance to travel and time taken for some participants to get to the gym were barriers to regular programme attendance. It would be difficult for some to attend if they did not have cars, if their cars broke down or if they had to rely on public transport, which was perceived to be expensive, resulted in longer travel times and could also be inefficient and unreliable. There were also other circumstances mentioned which limited attendance, for example holidays, and attending hospital appointments.

## 6 PERCEPTION OF SAFETY

It was important to participants to feel safe within the programme, and this referred to not being injured during exercise training. Some described how intimidating and unsafe it felt for them to come to terms with the reality of having to exercise in a gym, and how they had initially felt the first time exercising in a gym. Participants mostly attributed their perception of safety to regular support and supervision by exercise instructor, who was thought to make exercising fun and less daunting.

Being supervised is good. Because── you've always got that person if something did happen, you've got someone there. Otherwise you don't know what to do. (The exercise instructor) makes me feel safe (Amelia, P7).

The instructor's role included showing participants how to work all the equipment, answering questions and observing everyone during exercise training. For some participants, the support provided by the exercise instructor inspired confidence in their ability to subsequently exercise and use gym equipment with minimal or no supervision. In addition, support from peers helped people to feel safe when they needed assistance with the machines.

Participants also observed that they had learnt to take personal responsibility for own safety by adopting self‐protective measures to minimise risk of injuries and breathlessness while physically active, including not overdoing activity.

I can make my bed, but I have to pace myself. I can cook a meal, but I have to go at a certain pace. So, I’m still doing things, but I have to balance them and curtail and not go silly (Brendan, P23).

## 7 RECOMMENDATIONS FOR IMPROVEMENT

Finally, participants were asked how they thought the programme could be improved. The most frequently mentioned recommendation was having a purpose‐built unit with the proper sets of equipment for exercise. The reason given for this was because it would help them exercise in a less crowded place and use the correct equipment to work specifically on their chest. It was also thought it would enable flexibility, in terms of not having to always fit in with other classes scheduled at the gyms.

I don't see that you could── change that very much in the situation that it is, other than having a purpose built unit to go to, which has got the── proper equipment to suit our conditions and the space to do all the other work as well. There should be a unit dedicated to this. You want a proper unit with the proper equipment. Because according to (the exercise instructor), that's not the proper equipment we should be using. I mean, I would prefer more equipment that give you── more chest [sic] you know, to keep, it is the chest you are working on after all (Michael, P17).

Despite suggesting the provision of a purpose built facility, participants described how pleased they were with the existing programme and spoke very positively of the good organisation and programme components (time of exercise sessions, exercise modalities, frequency and duration). Regarding exercise modalities, eleven of the participants said they liked the studio breathing, seated warm‐up and cool down exercises better than using the gym equipment. Hence, they felt strongly that the seated warm‐up and cool down exercises should be continued. They intended to keep attending the group and suggested expanding the number of locations in which the programme was held for the benefit of others:

I think it [the exercise programme] works very well and I think that people at (Thompton Town) are lucky that they were there, but I don't know if everybody does it all over the place. It's good (Brendan, P23).

I think it's── more people ought to go to it who have breathing problems (Tony, P22)

The programme was considered essential for people with breathing problems. All participants wanted the programme to keep going and hoped it would not be cut because of pressures on budgets.

## 8 DISCUSSION

We conducted this study to explore perceptions and experiences of benefits, facilitators and barriers to participation in a CEP among people with COPD. Improvements in clinical outcomes associated with daily physical activity from attending the programme were reported elsewhere (Meshe, 2018). Regarding benefits of the CEP, our findings demonstrated that participants perceived the programme positively. They described several health, physical, psychological and social benefits of the programme. To our knowledge, only one previous research examined participants’ experiences of a CEP (Desveaux et al., 2014) and this was published while our study was in progress. In contrast to this previous research, which explored these issues by using focus groups involving 12 persons with COPD who attended a CEP, supervised by a physiotherapist, for 6 months, we used 1:1 semi‐structured interviews of 12 participants supervised by an exercise instructor for three months. Participants in the study by Desveaux et al., (2014) reported physical, psychological and social benefits that are consistent with our findings. What this present study adds is that, most participants emphasised the need for regular attendance in order to avoid deterioration in perceived benefits.

Preventing decline in benefits of PRPs is one of the goals of current guidance for COPD management (Vestbo et al., 2013). We identified that participants felt that physical ability and COPD symptoms deteriorated when they stopped attending the exercise classes for any reason. This result resonates with quantitative studies that reported decline in clinical outcomes following PR (Egan et al., 2012; Holland et al., 2017; Karapolat et al., 2007). To our knowledge, ours is the first qualitative study to corroborate such finding. It should be noted that the decline in benefits reported in quantitative studies was observed 1–12 months after cessation of the PRPs and this provided the evidence for the recommendation of CEPs by clinical guidance (Vestbo et al., 2013). Results from the present study indicate participants experience deterioration within an ongoing programme due to irregular attendance, emphasising the need to keep participants motivated and supported to overcome perceived barriers to attendance.

As with previous research (Desveaux et al., 2014), our study participants reported more facilitators than barriers to programme attendance, indicating a positive attitude to the programme. They expressed their desire for the programme to continue and a commitment to keep attending and also recommended it for people with similar conditions. Unlike Desveaux et al. (2014) study where participants’ recommendations for programme improvement mostly addressed the burdens associated with the programme (e.g. negative aspects of the overall design, access during winter months, cost and proximity), participants in this study did not describe these difficulties. They did however recommend having a purpose‐built facility with the suitable equipment for exercise maintenance, considering their age and conditions.

An important facilitator of attendance was ease of access to the CEP. In previous research, difficulty accessing a rehabilitation programme independently predicted poor attendance (Sabit et al., 2008). Many participants believed they attended regularly because the CEP was easily accessible. As with other research (Desveaux et al., 2014; Stewart et al., 2014), participants acknowledged that the CEP enabled them develop camaraderie and social support networks, which encouraged and sustained their regular attendance. In addition to the social benefits, our participants also believed that participating in the programme benefitted their physical and psychological health, which further motivated them to attend. This aligns with the ‘perceived benefits of taking action’ construct of the Health Belief Model (HBM) (Hochbaum, Rosenstock, & Kegels, 1952), whereby if an individual believes a particular action will decrease seriousness of a condition they are more likely to engage in that action or activity.

Our study identified five barriers to attendance, which are similar to those highlighted in previous studies (Hellem, Bruusgaard, & Bergland, 2012; Keating, Lee, & Holland, 2011; Thorpe, Johnston, & Kumar, 2012). Deterioration in physical health was the most frequently cited barrier. This is expected in people with moderate to severe COPD given the chronicity of the disease and its negative impact as well as the fact that rehabilitation programmes cannot completely stop the progressive deterioration in COPD symptoms. Another key barrier was transport difficulties, which were mostly mentioned by four participants who lived about ten miles away from the gym. Transport difficulties was linked to inability to attend if they did not have cars, if their cars broke down or if they had to rely on public transport, which is expensive and sometimes unreliable. The latter is an important factor, because although people may be motivated to join a physical activity group, it is their ability to travel easily to exercise venues facilitated through access to a car or public transport, which maintains attendance (Keating et al., 2011). Addressing barriers will be required to ensure regular attendance, which will in turn reduce deterioration in benefits of rehabilitation programmes. Participants who perceived fewer barriers to their participation in CEPs will be more likely to attend, again in line with the ‘perceived barriers to action’ construct of the HBM (Hochbaum et al., 1952).

In contrast to the research by Desveaux et al. (2014), our findings draw attention to motivation in relation to increasing self‐confidence, self‐efficacy and empowerment. Hogg et al. (2012) described self‐confidence as ‘strength of belief’. It is a central part of perceived self‐efficacy, which refers to the belief in one's ability to carry out a particular task (Bandura, 1997). People with COPD have low self‐efficacy for coping with breathlessness associated with performing physical activities (Wigal, Creer, & Kotses, 1991). According to Troosters et al. (2013), fear of breathlessness is a common problem for people with COPD and it is a major factor for physical inactivity. Our findings suggest that attending CEP can redress this undesirable influence. In agreement with Bandura's theoretical sources of self‐efficacy (Bandura, 1997), most participants explained how they derived motivation from verbal persuasions and encouragement by their peers and exercise instructor and associated this with self‐efficacy for coping with breathlessness and participating in PA.

Self**‐**efficacy and locus of control are related constructs. The later refers to the degree to which an individual feels or believes s/he has control over the outcome of an event (Rotter, 1966). Importantly, participants demonstrated positive beliefs in their ability to control adverse outcomes such as fatigue, discomfort and breathlessness associated with engaging in physical activities within the CEP, reflecting internal locus of control (Rotter, 1966). The positive beliefs and internal locus of control positively influenced self‐confidence and perceived ability to complete exercise modalities as well as facilitated regular attendance. Our data support the element of the HBM (Hochbaum et al., 1952), which predicts a positive impact of high self**‐**efficacy through engagement with health‐enhancing behaviour e.g. attending a CEP.

We also identified that the presence of the exercise instructor was key, providing a sense of feeling safe, and to people experiencing no complications during exercise performance. Concerns about personal safety were previously cited as barriers to attending a CEP (Desveaux et al., 2014), leading to participants suggesting several recommendations on how to enhance safety. In contrast, our findings highlighted that participants reported feeling safe in this programme and linked this to being regularly supported and supervised by the instructor.

### 8.1 Limitations

The 12 participants in this study were recruited from a single CEP and were White British and retired from full‐time paid work. It is not known if ethnicity and work status played a role in our findings and could be an area for future research. This study needs replicating, it is possible that people with COPD in other programmes will experience benefits, barriers and facilitators differently. All participants had previously completed initial PR, therefore, it is not appropriate to apply our results to settings where people with COPD enter home or community‐based fitness programmes without having previously completed PR.

## 9 CONCLUSION

In summary, our study showed that participating in a CEP was associated with physical, social and psychological benefits. Participants perceived that exercising in a group setting motivated them to adhere to exercise training. They perceived regular attendance as relevant to preventing deterioration in benefits of the programme. Furthermore, participants identified factors that enabled and prevented regular attendance. These findings have relevance for people in the post‐PR phase of COPD management and provide clues on how to achieve the goals of current guidance for COPD management, particularly in relation to preventing relapse to insufficient levels of daily PA and decline in benefits of PRPs (Vestbo et al., 2013; Watz et al., 2014).

To address the barriers to attending regular CEP, service providers are encouraged to work closely with people with COPD to involve them in decisions about their care and understand whether they are at risk of non‐completion to existing CEPs. Addressing transportation difficulties will require expanding the number of suitable low‐cost CEP, in order to facilitate access and reduce the burden of travel. For people to attend CEPs regularly requires empowering participants to take control of their lives and act in a more healthy way (Rotter, 1966). When improving existing CEPs or developing new ones, it is essential to embed strategies to increase participants’ self‐efficacy and internal locus of control through, for example scheduled time for social interaction to share vicarious experiences and develop peer support opportunities.
